# Clinical and imaging features of reversible splenial lesion syndrome with language disorder

**DOI:** 10.1515/tnsci-2020-0126

**Published:** 2020-06-19

**Authors:** Yi Tang, Dong Zhang, Jian Ge, Jing Jin, Yumeng Liu, Siyuan Chen, Mingli He

**Affiliations:** Department of Neurology, The affiliated Lianyungang Hospital of Xuzhou Medical University, Tongguan North Road, No. 182, Haizhou District, Lianyungang, Jiangsu, China

**Keywords:** reversible splenial lesion syndrome, corpus callosum, splenium of the corpus callosum, cognitive impairment, language impairment

## Abstract

Reversible splenial lesion syndrome (RESLES) is a single-stage non-specific syndrome with unclear pathogenesis. There has been no report on answer delay in patients with RESLES. We report a female patient who was admitted to our department for mixed aphasia accompanied by cognitive impairment. During the rapid improvement of aphasia, there was a clear phase of language output response delay accompanied by resolution of imaging lesions. We analyzed the course and the examination results of the patient and speculated the cause and pathogenesis. RESLES-relevant knowledge was systematically reviewed, which will help doctors in the classification of cerebral function and the diagnosis of RESLES. The specific language and cognitive impairment may be associated with the damage of contact fibers in the bilateral primary and secondary sensory and motor cortices.

## 1 Introduction

Reversible splenial lesion syndrome (RESLES) is a clinical–imaging syndrome caused by various factors affecting the corpus callosum. It is characterized by reversible round or oval, non-enhanced lesions in the corpus callosum on magnetic resonance imaging (MRI). According to the characteristics displayed on MRI, RESLES is classified as type I, only involving the splenium of the corpus callosum, and type II, involving the white matter and/or whole carcass [1]. The pathological basis is cytotoxic edema of the corpus callosum, which is caused by water trapped within astrocytes and neurons. The receptors’ density of the nerve cells in the corpus callosum is high, which easily promotes the development of cytotoxic edema when cytokinopathy occurs [2]. The clinical manifestations include disturbance of consciousness (65.9%), convulsions (43.2%), dysarthria (18.2%), hallucinations (9.1%), ataxia (9.1%), headache (6.8%), and dizziness (6.8%) [3]. Herein, we report a patient with RESLES who presented with language disorder and cognitive impairment. The characteristic manifestation of this patient’s language disorder is a delayed answer. It is not only rare in RESLES but also in other diseases that could cause local neurological deficits, and we think it is related to nerve fiber damage.

## 2 Case report

### 2.1 Patient’s medical history

A 54-year-old female patient, junior high school education level, was admitted to the hospital with intermittent watery diarrhea for 7 days and progressive speech disorder for 3 days. The cranial computed tomography showed no abnormality at the community hospital and she was immediately referred to the hospital. There were no symptoms of numbness, fatigue, disturbance of consciousness, limb convulsions, urinary incontinence, and fever since the onset and no family history or previous history of neurological disorders. However, due to the swelling of the buttocks for nearly 20 days, the patient applied self-purchased drugs on bedsore wounds in the hip area, while receiving an intravenous infusion of penicillin.


**Informed consent:** Informed consent has been obtained from the patient who participated in this study.
**Ethical approval:** The research related to human use has been complied with all the relevant national regulations and institutional policies and performed in accordance with the tenets of the Declaration of Helsinki and has been approved by the authors’ institutional review board or equivalent committee.

### 2.2 Tests on admission

On admission, she was conscious and silent, with a temperature of 36.8°C. The patient’s visual space and executive ability, attention, abstract thinking, and short-term memory were significantly impaired. Her answers to questions were delayed by 8 s, even after multiple repetitions. The Montreal Cognitive Assessment (MoCA) score was 3 points, and the normal value range is not less than 26 points.

The patient’s right leg was disabled, with severe weakness and pathological reflex. The right upper limb had an increased tone, whereas other neurological examinations showed no abnormality. Leukocyte counts were 10.75 × 10^9^/L, the ratio of neutrophils was 91.8%, and the proportion of lymphocytes was 4.5%. The patient was positive for herpes simplex virus IgG antibodies and a titer of 161.10 U/mL for cytomegalovirus IgG antibodies; serum potassium concentration was 3.2 mmol/L. Renal function, liver function, routine stool test, blood culture for bacterial infection, erythrocyte sedimentation rate, D-dimer, electrolytes, cerebrospinal fluid, and other related examination results were normal. The electroencephalogram showed an extensive and mild abnormality of the adult type, and the slow-wave band value was high.

### 2.3 MRI observations

**Figure 1:**
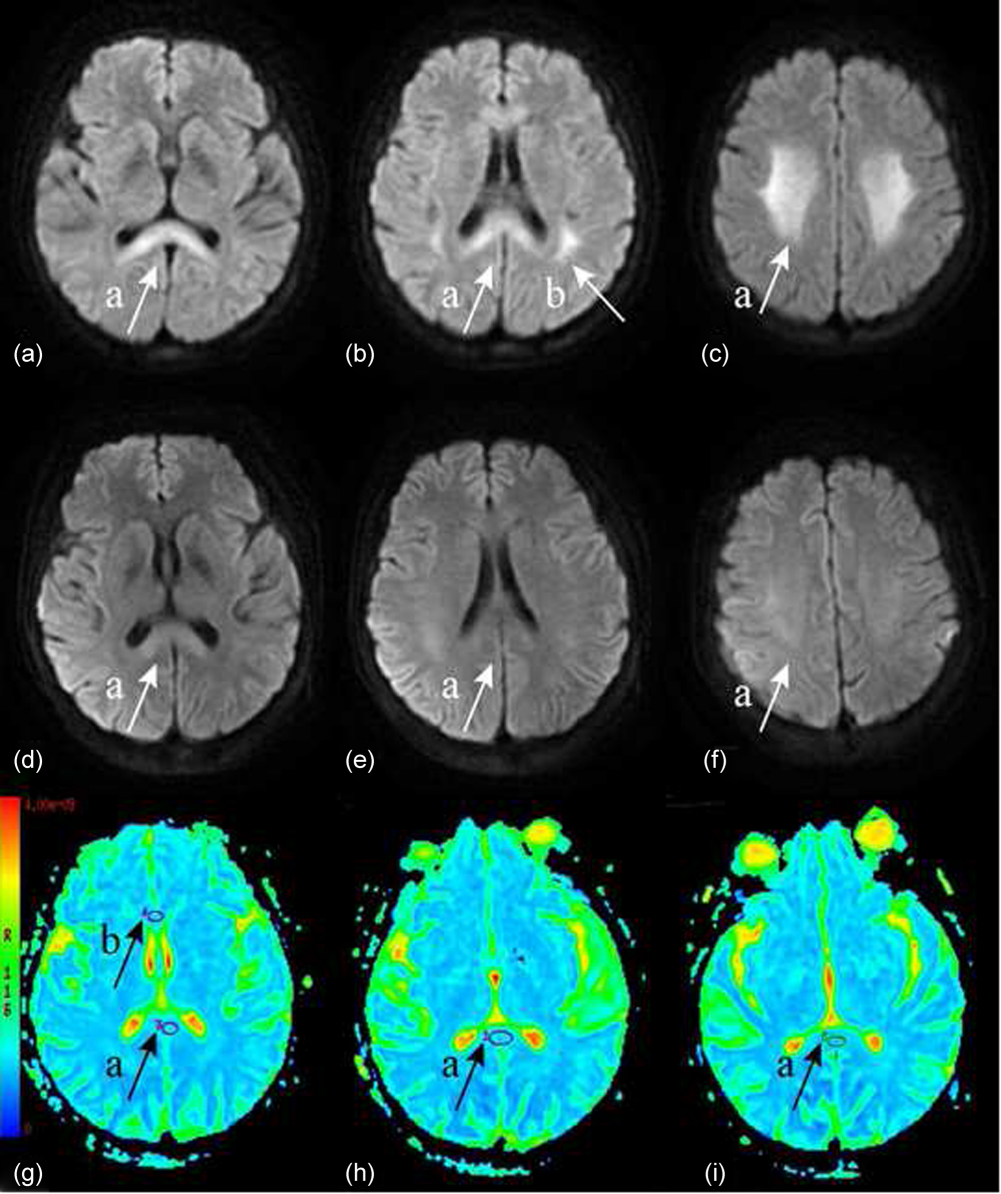
Magnetic resonance images. (a-a/b-a) DWI of the corpus callosum. The signal is limited, showing a “boomerang sign” on day 9; (b-b) DWI showing high signal shadows in the lateral ventricle on day 9; (c-a) DWI showing limited signal in the bilateral semioval center on day 9; (d-a/e-a) DWI showing that the high signal in the corpus callosum has basically disappeared. (f-a) The bilateral semi-oval center lesions are significantly reduced. (g-a/h-a/i-a) The region of interest (ROI) in the splenium of the corpus callosum. (g-b) The genu of the corpus callosum as a control.

On day 9, we found decreased intensity of bilateral basal ganglia lesions on T1-weighted (T1W) images and increased signal intensity on T2-weighted (T2W) images and fluid-attenuated inversion recovery (FLAIR). The splenium of the corpus callosum demonstrated relatively decreased signal intensity on T1W images and increased signal intensity on T2W images. Its signal intensity was slightly higher than that in normal brain tissue on FLAIR. Diffusion-weighted imaging (DWI) showed hyperintensity of it resembling *boomerang sign*. The genu of the corpus callosum also showed relatively high signal intensity on DWI (Figure 1a and b). Bilateral periventricular white matter lesions and centrum semiovale showed high signal intensity on DWI and FLAIR (Figure 1c). On day 16, DWI showed a complete resolution of lesions in the centrum semiovale, and lesion in the splenium had decreased in size and signal intensity (Figure 1d–f). Moreover, diffusion tract imaging showed that the nerve fiber bundle was normal. The mean apparent diffusion coefficient (ADC) value of the splenium increased from 0.476 × 10^−3^ to 0.830 × 10^−3^ mm^2^/s. The mean ADC value of the genu increased from 0.694 × 10^−3^ to 0.861 × 10^−3^ mm^2^/s (Figure 1g and h).

### 2.4 Clinical course and outcome

According to the patient’s status at the time of admission, the initial diagnosis was central nervous system infection, and the patient was given antiviral and dehydration treatments during hospitalization. From the ninth day after the onset, the patient’s symptoms, including the mixed aphasia, rapidly improved, and the diagnosis was corrected based on the above-mentioned magnetic imaging features.

Sixteen days after the onset, significant recovery was observed in all cognitive subdomains, she answered questions in 4 s, and the MoCA score was 7 points. Twenty-eight days after the onset, the cognitive subdomain impairments were restored to normal, she responded to questions in 1 s, and the MoCA score was 21 points.

## 3 Discussion

In 1999, Kim et al. first reported reversible corpus callosum lesions [4]. In 2004, Tada et al. proposed the concept of “mild encephalitis/encephalopathy with a reversible splenial lesion (MERS)” [5]. Subsequent studies have shown that although MERS can well explain reversible corpus callosum lesions in children, it is not fully applicable to adults. Garcia-Monco et al. described this syndrome in detail based on previous studies and proposed a new term RESLES in 2011 [6].

Although patients with RESLES have no specific clinical symptoms, answer delay is rare even in other diseases. Patients with RESLES often have prodromal symptoms, including fever and diarrhea, followed by seizures, confusion, ataxia, lethargy, headache, paralysis, etc. [7]. RESLES is primarily diagnosed based on imaging features and clinical presentations. Accordingly, some researchers have proposed the following diagnostic criteria for RESLES: (1) mild central nervous system damages, such as paralysis and mild disturbance of consciousness; (2) high signal in DWI of the splenium of the corpus callosum; and (3) rapid relief and full recovery of symptoms within 1 month [5]. Due to the atypical clinical symptoms, the most important means of MERS diagnosis is MRI. The clinical manifestations and imaging features of the present case completely met the above-mentioned criteria. Takanashi et al. divided MERS into two types based on whether the lesion was confined in the splenium of the corpus callosum. The prognosis for RESLES type I is often better than that of type II [1]. However, some findings suggest that MRI may reveal a longitudinal change from type II to type I. The radiological features of type I could also be a part of the course of type II [8].

RESLES should be differentiated from diseases such as acute cerebral infarction, acute disseminated encephalomyelitis, and extrapontine myelinolysis. The symptoms of the case that we report were self-limiting and imagings revealed a reversible lesion resembling the boomerang sign that distinguishes it from other diseases and should be classified as type II. Most of the earliest cases were patients with epilepsy or infants. Due to the widespread use of MRI, various cases associated with drug, malignancy, infections, subarachnoid hemorrhage, metabolic abnormalities, and trauma have been emerged [2]. A few cases with high-altitude cerebral edema [9], poisoning [10], vaccination [11], Kawasaki disease [12], and postpartum cerebral angiopathy [13] were also found to suffer from RESLES. Most cases were infection-induced RESLES. The woman we report has no history of epilepsy, high-altitude cerebral edema, hypoglycemia, electrolyte disorder, or obvious malnutrition. Diarrhea and routine blood tests revealed probable gastrointestinal infections; however, we did not find enough evidence of recent infection. No pathogen was found in feces or blood, even in cerebrospinal fluid. It may be caused by previous inappropriate antibiotic use. In addition, what we could not exclude is that her self-purchased drugs would have contributed to RESLES. The specific ingredients of the drug have not been indicated. We were not able to analyze its composition due to the limited conditions. Drugs reported leading to RESLES like steroid hormones, are likely to be included. Until the patient asked to be discharged, regrettably, we have been unable to formulate the explicit etiology.

The pathogenesis of RESLES is not clear. Various pathogenic factors yield the same imaging features indicating that these pathological mechanisms must involve some common factors that should be explored. Starkey et al. proposed that the mechanism of cytotoxic lesions of the corpus callosum is the excitotoxic action of glutamate on *N*-methyl-d-aspartate receptors, sodium–potassium pumps, etc., which results in water flow into astrocytes and neurons [2]. The density of fiber in the corpus callosum, and particularly the splenium is high, which results in high density of receptors leading to a tendency for cytotoxic edema of the splenium. Starkey et al. classified cytotoxic lesions of the corpus callosum into the following three categories: (1) a small lesion in the center of the splenium of the corpus callosum which is usually round or oval; (2) the lesion is centered in the splenium of the corpus callosum, but extends outward through the corpus callosum fibers into the adjacent white matter; and (3) the lesion is centered on the back of the corpus callosum and extends to the front of it [2]. Other theories implicate transient inflammatory responses [14] and genetic factors in the pathogenesis of RESLES [15].

The corpus callosum, located at the bottom of the fissura longitudinalis cerebri, is a transverse nerve fiber bundle connecting the left and right hemispheres of the brain. It plays an important role in the transmission and integration of writing, body, face, and visual information. Therefore, our patient’s silent state on admission may be caused by the disconnection of the two hemispheres and the disruption of cerebral cortex functioning [16]. After the improvement of mixed aphasia, although the patient showed an obvious delay in communication, her responses were rapid, as a blank space was inserted into the normal conversation. She could answer simple questions correctly, and her symptoms were similar to those of parietal aphasia [17]. There was no obvious left hemiparalexia or left hemialexia. She was unable to comprehend the information in sentences. The above-mentioned concurrent symptoms indicated that the patient may have a disorder in semantic activation and integration [18]. In the MoCA test, the patient could not combine or arrange the graphics and could only draw two-dimensional graphics. In recent years, some researchers have found that brain white matter lesions in the corpus callosum are significantly associated with the speed of sports-related information processing in patients, although this correlation has not been confirmed by other studies and still needs to be further explored [19]. The cognitive manifestations of our patient reflect the intrinsic mechanism of contact fiber impairment in the bilateral primary and secondary sensory and motor cortices in RESLES.
